# A Crucial Role of IL-17 and IFN-γ during Acute Rejection of Peripheral Nerve Xenotransplantation in Mice

**DOI:** 10.1371/journal.pone.0034419

**Published:** 2012-03-30

**Authors:** Xin Yu, Yanfang Jiang, Lu Lu, Xu Gong, Xiguang Sun, Zhaopeng Xuan, Laijin Lu

**Affiliations:** 1 Department of Hand Surgery, First Hospital, Jilin University, Changchun, China; 2 Department of Central Laboratory, the Second Part of First Hospital, Jilin University, Changchun, China; University Paris Sud, France

## Abstract

Nerve injuries causing segmental loss require nerve grafting. However, autografts and allografts have limitations for clinical use. Peripheral nerve xenotransplantation has become an area of great interest in clinical surgery research as an alternative graft strategy. However, xenotransplant rejection is severe with cellular immunity, and Th1 cells play an important role in the process. To better understand the process of rejection, we used peripheral nerve xenografts from rats to mice and found that mononuclear cells expressing IFN-γ and IL-17 infiltrated around the grafts, and IFN-γ and IL-17 producing CD4+ and CD8+ T cells increased during the process of acute rejection. The changes of IL-4 level had no significant difference between xenotransplanted group and sham control group. The rejection of xenograft was significantly prevented after the treatment of IL-17 and IFN-γ neutralizing antibodies. These data suggest that Th17 cells contribute to the acute rejection process of peripheral nerve xenotransplant in addition to Th1 cells.

## Introduction

Peripheral nerve injuries are very commonly seen in the clinic. If they cannot be immediately repaired to recover continuity and integrity, they often result in motor and sensory deficits for patients [1]. In many instances these injuries result in long nerve segment loss preventing the nerve ends from being directly sutured due to increased tension [2]. In these cases grafts such as autografts and allografts must be used to bridge the long nerve gaps. Although autografts are recognized as the gold standard for nerve grafting, they have several limitations including limited donor source, functional loss in the distribution of the donor nerve, scarring, and painful neuroma formation [3]–[4]. Allografts also have defects in limitations of source [5]. In order to find a means of reconstructing injured nerves, peripheral nerve xenotransplantation has become an increasingly promising alternative.

A significant barrier to xenotransplantation is the humoral and cellular rejection that can occur which is mediated by antibodies, T cells, and innate immune cells. Cellular immune responses that play a critical role in nerve graft rejection [6]–[7], are likely to be more important than humoral immune responses. For instance, human T-cell responses against pig antigens are stronger than against alloantigens *in vitro*, limiting dilution analyses [8]. A study using a human complement regulatory protein (CD46)-transgenic pig-to-baboon heart transplantation model, in which the recipients were treated with a B-cell-depleting antibody and an α1,3-galactosyltransferase (α1,3Gal) polymer to remove α1,3Gal-specific antibodies, but not with anti-T-cell therapy, revealed an important role for T cells, in particular, in xenograft rejection [9]. In our previous study using mouse peripheral nerve xenografts in a rat model, we also demonstrated that a transient and moderate increase in the production of Th1 cytokines, including IL-2, IFN-γ, and TNF-α occurs during rejection, but there were no significant changes of Th2 cytokines after xenograft transplantation, such as IL-4 [6]. Therefore, Th1 cells and the cytokines they produce, likely play an important role in rejection during mouse peripheral nerve xenotransplantation.

Classically, effector CD4+ T cells have been assigned to the Th1 or Th2 lineage based on their cytokine profiles. Th1 cells evolved to enhance clearance of intracellular pathogens, and are defined on the basis of their production of IFN-γ. Th2 cells are critical for the control of certain parasitic infections through the production of the clustered group of cytokines, IL-4, IL-5 and IL-13. The Th1/Th2 paradigm has been used to identify the mechanisms of different organ transplant rejections. The concept involves the notion that the two subsets are cross-regulated. Thus, cytokines released from cells of one subset have the ability to stimulate their own subset in an autocrine fashion and, at the same time, inhibit the other subset.

Recently, these two classical lineages have been joined by additional subsets that preferentially produce distinct cytokines. One such subset is called Th17, which selectively produces IL-17 [10]–[15]. Th17 cells are important for clearance of a variety of pathogens [16], and are associated with numerous autoimmune and inflammatory conditions. Th17 cells have also been implicated in acute and chronic rejection in animal models of transplantation [17]–[20]. Just as Th1 and Th2 responses have long been thought to regulate one another, there is similar evidence regarding the relationship between Th1 and Th17 cells. Lack of one of these cytokines *in vivo* appears to promote a response dominated by the other [14], [21]–[24]. However, they are not always functionally antagonistic, but may in fact collaborate to accomplish some effector functions [25]–[30].

The role of Th17 cells and IL-17 in peripheral nerve xenotransplantation has not yet been defined. We therefore, again used the rat to mouse model to investigate the biological importance of Th17, and the relationship between Th1 and Th17 cells in peripheral nerve xenotransplant rejection. We found that the changes of IL-4 level had no significant difference in xenograft recipients,which is consistent with our previous result [6]. While the mononuclear cells which expressed IFN-γ and IL-17 infiltrated around the grafts and IFN-γ and IL-17 producing CD4+ and CD8+ T cells significantly increased during the acute rejection process. Furthermore, the rejection was prevented after the treatment of IL-17 and IFN-γ neutralizing antibodies. These data indicate that Th17 cells are also involved in the process of peripheral nerve xenotransplant acute rejection, in addition to classically described Th1 cells.

## Materials and Methods

### 1. Animals

Adult male Sprague Dawley rats (weighing 190 g) and adult male BALB/c mice were used as donor and recipients, respectively (4 mice at least in each group). All animals were purchased from Jilin University (Changchun, China). All surgical procedures and postoperative care of the animals were approved by the Institutional Animal Ethics Committee.

### 2. Peripheral Nerve Xenotransplantation

The rat sciatic nerve of the donor was exposed through a dorsal gluteal muscle splitting incision, and a segment (0.5 cm) of sciatic nerve was harvested and used immediately. The skin over the mouse recipient right hindlimb was incised, the muscle was bluntly dissected to expose the sciatic nerve, and a 0.5 cm gap was created. The rat sciatic nerve graft was interposed to the transected nerve and immediately repaired with 11-0 nylon epineurial sutures. Mice in which the sciatic nerve was exposed without xenotransplantation, were used as the sham control group. Eight mice (4 from the xenotransplant group and 4 from the sham control group) were sacrificed at each time point (Day 1, 3, 6, 11, 16, and 30).

### 3. IFN-γ and IL-17 neutralization *in vivo*

4 ug IFN-γ neutralizing antibody and 4 ug IL-17 neutralizing antibody (eBioscience, San Diego, USA) were injected respectively into peritoneal cavity of mice one day before xenotransplantation.

### 4. Histological Evaluation

Nerve grafts and the surrounding muscle and adipose tissues from sacrificed animals were fixed in 10% (v/v) neutral buffered formalin, and embedded in paraffin and sectioned. The 4-µm sections were stained with hematoxylin and eosin (H&E) for histological examination. The degree of mononuclear cells infiltration were scored in the section using a 200× objective and scored as follows: 1 = minimal, 2 = mild, 3 = moderate, 4 = severe pathology. This scale contains 0–100, 100–300, 300–600 and >600 cells respectively.

### 5. Immunohistochemical Staining

Anti-mouse IFN-γ antibody (XMG1.2, Westang Biotechnology, Shanghai, China), anti-mouse IL-17 antibody (Tc11-18h10, Westang Biotechnology, Shanghai, China), anti-mouse IL-4 antibody (BVD4-1D11, Westang Biotechnology, Shanghai, China) and rabbit IgG (Westang Biotechnology, Shanghai, China) were used for immunohistochemical staining of the nerve grafts. Four sections, which included the grafts and surrounding tissues, were stained for each sample, and three sections were randomly chosen to be stained with IFN-γ, IL-17 and IL-4. Tissue sections were cut from paraffin-embedded tissue blocks and placed onto slides. After deparaffinization, sections were soaked in target retrieval buffered saline (TRS, pH 6.1, Dako Cytomation, Carpinteria, CA) in a plastic pressure cooker containing no metals, and irradiated in a microwave oven for 10 minutes (maximum 500 W). After irradiation, sections were rinsed under running water for 2 minutes, soaked in 3% H_2_O_2_ methanol solution for 5 minutes, and then soaked in 5% BSA for 1 minute. Primary antibodies were diluted to a previously determined optimal concentration in PBS containing 5% BSA. The diluted antibodies were applied to the tissue sections in a humid chamber and irradiated intermittently for 10 minutes (250W, 4 seconds-on, 3 seconds-off). After 3 washes with Tris-buffered saline containing 1% Tween (TBS-T) for 1 minute, the peroxidase-conjugated Envision kit reagents for mouse (Westang Biotechnology, Shanghai, China) were applied to the appropriate specimens in the humid chamber. Irradiation was then performed intermittently for 10 minutes, as described above. After washing 5× with TBS-T, the sections were immersed in DAB solution (Sigma-Aldrich) with H_2_O_2_ and counterstained with Hematoxylin (Dako Cytomation) and mounted under coverslips. The degree of IFN-γ+, IL-17+, and IL-4+ cellular infiltration were scored in the section using a 200× objective and scored as follows: 1 = minimal, 2 = mild, 3 = moderate, 4 = severe pathology. This scale contains 0–15, 15–50, 50–100, and >100 positive cells respectively.

### 6. Enzyme-linked Immunosorbent Assay (ELISA)

Whole blood was collected and allowed to clot at room temperature for 20 minutes. The clots were removed by centrifuging at 1500×g for 10 minutes in a refrigerated centrifuge. The levels of cytokines in the serum supernatant were measured by ELISA assay using commercially available IFN-γ, IL-17 and IL-4 ELISA kits (Westang Biotechnology, Shanghai, China) according to the manufacturer's instructions.

### 7. Flow Cytometric Analysis of Intracellular Cytokine Staining (ICS)

Whole spleens were homogenized in PBS, and the cell suspension was filtered to remove debris. The cells were collected by centrifugation at 1500×g for 10 minutes in a refrigerated centrifuge. The cells were then washed and resuspended with FACS buffer. After processing, 1–2×10^6^ cells/mL were stimulated with phorbol 12-myristate 13-acetate (PMA; Sigma Chemical Co., St. Louis, MO) (50 ng/mL) plus 2 µg/ml of ionomycin for 6 hours. Brefeldin A (10 µg/ml) was added 2 hours before the cells were collected and stained with antibodies. The cells were stained with antibodies against surface markers (anti-mouse CD3 and CD8, eBioscience, San Diego, USA). Cells were then permeabilized and fixed using fixing (Caltag, Burlingame, USA) and membrane permeabilizing (Caltag, Burlingame,USA) reagents according to the manufacturer's instructions, followed by incubation with anti-IL-17-FITC (eBioscience, San Diego, USA), anti-IL-4-FITC (eBioscience, San Diego, USA), anti-IFN-γ-PE (eBioscience), or isotope-matched control antibodies for 30 minutes. Flow cytometry (FACSCalibur™, Beckton Dickinson) was performed and data were analyzed using FlowJo software.

### 8. Statistical Analysis

The difference between the means of the groups was determined by the student's t test for parametric data sets. All statistical analyses were performed using Prism 4 (GraphPad Software, San Diego, CA). A p value ≤.05 was considered statistically significant in all analyses herein.

## Results

### 1. Mononuclear cell infiltration surrounding the grafts

**Figure 1:**
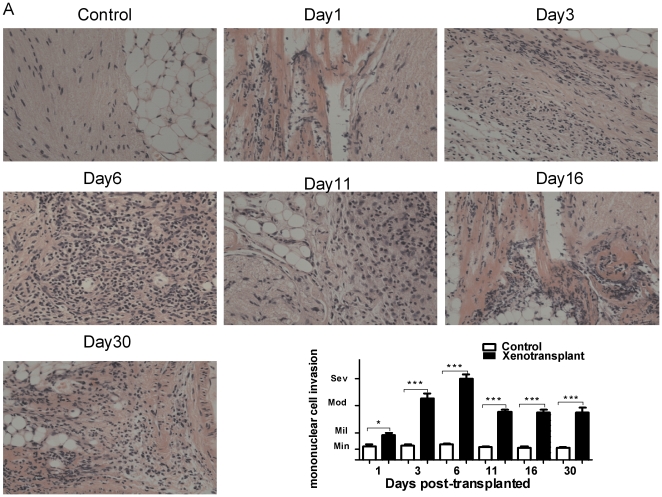
H&E staining of nerve grafts. Sham control or xenotransplanted sciatic nerve from mice was collected at 1, 3, 6, 11, 16, and 30 days posttransplantation, sectioned, and stained with H&E. Staining demonstrated xenograft adhesion to the surrounding tissue, and infiltration of mononuclear cells into the peripheral nerve and surrounding tissue. There was no significant difference during 30 days after xenotransplantation in control group. The level of mononuclear cell infiltration, distension, and necrosis significantly increased in all xenografts at all time point after transplantation compared with control group. Magnification: 200×. (We performed animal experiment twice, and stained 2 sections for each animal.)

In order to examine the general cellular changes around the grafts during rejection, the tissue was collected, sectioned and visualized using routine H&E staining. Compared to the control group, we observed that xenograft adhesion to the surrounding tissue was progressively overwhelmed in all mice during the 30-day observation period in all grafted animals. Histologic evidence of rejection was identified in all grafted tissue samples. There was no significant difference during 30 days after xenotransplantation in control group. The level of mononuclear cell infiltration, distension, and necrosis significantly increased in all xenografts at different time point after xenotransplantation compared with the control group. (Fig. 1).

### 2. IFN-γ, IL-17 and IL-4 immunoreactivity around the grafts

**Figure 2:**
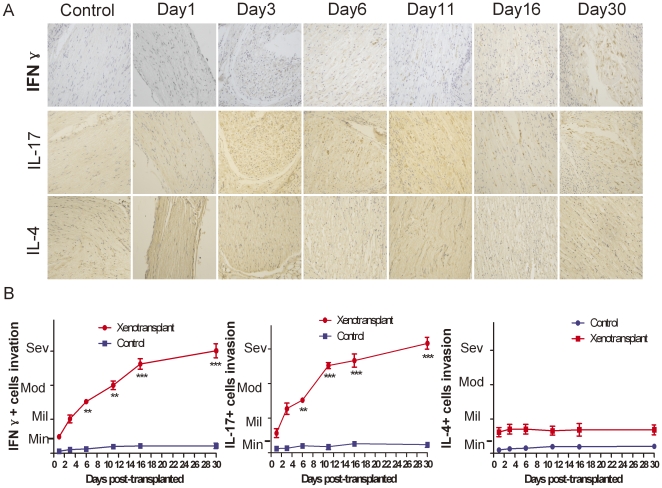
Immunohistochemical staining of nerve grafts. (A) Immunohistochemical staining of collected nerve grafts from sham control and xenotransplanted mice, were immunostained using anti-IFN-γ, anti-IL-17, and anti-IL-4 antibodies with DAB as the chromogen with a Hematoxylin counterstain. Both antibodies demonstrated scattered immunoreactivity in the infiltrating mononuclear cells. Representative images are shown. Magnification: 200×. (B) IFN-γ+ cells and IL-17+ cells increased in xenotransplanted group from day 6 to day 30 compared to the sham controls. There is no significant difference of IL-4+ cells between the recipients and control group. *, p<0.05; **, p<0.01; *** p<0.001. (We performed experiment twice, and stained 2 sections for IFN-γ and IL-17 respectively of each animal.)

In order to determine whether the mononuclear cells surrounding the xenografts expressed IFN-γ, IL-17, and IL-4, we next performed immunohistochemical staining. We found that scattered immunoreactivity for IFN-γ, IL-17 and IL-4 can be detected in the infiltrating mononuclear cells in the recipients animals compared to the sham controls. Evenmore, IFN-γ+ cells and IL-17+ cells significantly increased in the xenografts recipients between day 6 and day 30. In contrast, no significant changes of IL-4+ cells in the tissues surrounding the xenograft have been dectected. (Fig. 2).

### 3. IFN-γ and IL-17 levels transiently increased in sera during acute rejection

**Figure 3:**
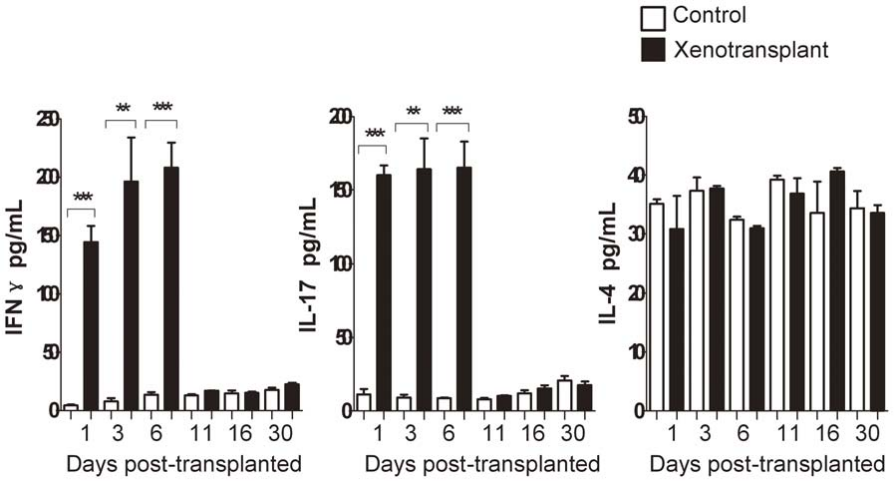
ELISA assays of IFN-γ, IL-17 and IL-4 in serum. Sera from collected blood at each time point (1, 3, 6, 11, 16, and 30 days) from xenotransplanted and sham control mice were used for ELISA analysis. Sera IFN-γ and IL-17 levels in the xenograft group were significantly higher than those in the serum in the control group (p<0.05) at day 1, day 3 and day 6, and decreased to control levels by day 11. There is no significant difference of IL-4 levels between recipients and control group. *, p<0.05; **, p<0.01; *** p<0.001. (We performed this experiment twice).

To provide a more quantitative assessment of changes in IFN-γ and IL-17 during rejection, concentrations of both cytokines were quantified by ELISA from sera of xenograft and sham control animals. The sera levels of IFN-γ and IL-17 at day 1, 3, and 6 were statistically higher in the xenograft recipients compared to the control group. The levels of both cytokines returned to control values by day 11 after transplantation. The IL-4 levels in sera did not show significant changes between the recipients with xenograft transplantation and the control group (Fig. 3).

### 4. IFN-γ and IL-17 expression in spleen lymphocytes transiently increased during acute rejection

**Figure 4:**
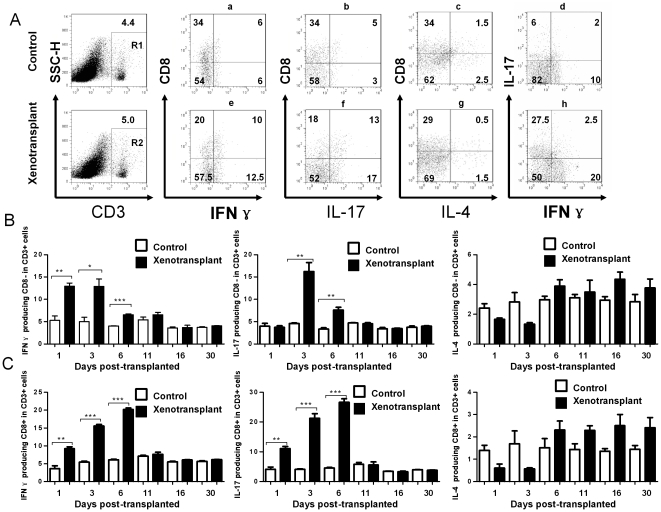
Flow cytometry analysis of IFN-γ, IL-17 and IL-4 in lymphocytes from spleen. Spleens were collected at each time point from xenotransplanted mice (day 1, 3, 6, 11, 16, and 30) and sham controls, and used for flow cytometry analysis (A) Representative data of IFN-γ, IL-17 and IL-4 expression in lymphocytes from spleen at day 1. Graph a, b, c and d are gated from R1; e, f, g, and h are gated from R2. (B) Percentage of CD3+CD8− T cells expressing IFN-γ, IL-17 and IL-4 at days 1–30 were averaged and graphed. IFN-γ expression increased from day 1 to day 6, and decreased to control levels by day 11. IL-17 expression increased at day 3 and lasted until day 6, and decreased to control levels by day 11. There is no significant difference of IL-4 producing CD3+CD8−T cells between recipients and control group. *, p<0.05; **, p<0.01; *** p<0.001. (C) In CD3+CD8+ T cells, IFN-γ and IL-17 expression increased from day 1 to day 6, and decreased to control levels by day 11. There is no significant difference of IL-4 producing CD3+CD8+T cells between recipients and control group. *, p<0.05; **, p<0.01; *** p<0.001. (We performed this experiment twice).

In order to better characterize the cellular identity of the IFN-γ, IL-17 and IL-4 expressing cells, spleens were collected from the mice at each time point and used for flow cytometry to validate expression of IFN-γ, IL-17 and IL-4 in lymphocytes after transplantation (Fig. 4A). IFN-γ and IL-17 double producers from CD3+ cells were fewer than single producers, and there was no significant difference between experimental and control group after xenotransplantation (data not show). We found that IFN-γ producing CD3+CD8+ T cells and IL-17 producing CD3+CD8+ T cells significantly increased after transplantation. Similar to the changes observed in sera, there were no differences between the sham control and xenotransplant groups after day 11 (Fig. 4B–C). IFN-γ producing CD3+CD8− T cells significantly increased from day 1 which lasted to day 6, but IL-17 producing CD3+CD8− T cells demonstrated a delay and increased from day 3 which lasted to day 6. The percentage of IFN-γ producing CD3+CD8− T cells and IL-17 producing CD3+CD8− T cells demonstrated no differences between the xenotransplant group and sham control group after day 11 (Fig. 4B–C). There are no significant changes of IL-4 producing CD3+CD8−T cells and IL-4 producing CD3+CD8+T cells between xenografts recipients and sham controls. (Fig. 4B–C)

### 5. The rejection of peripheral never xenografts significantly alleviated after using IL-17 and IFN-γ neutralizing antibodies

**Figure 5:**
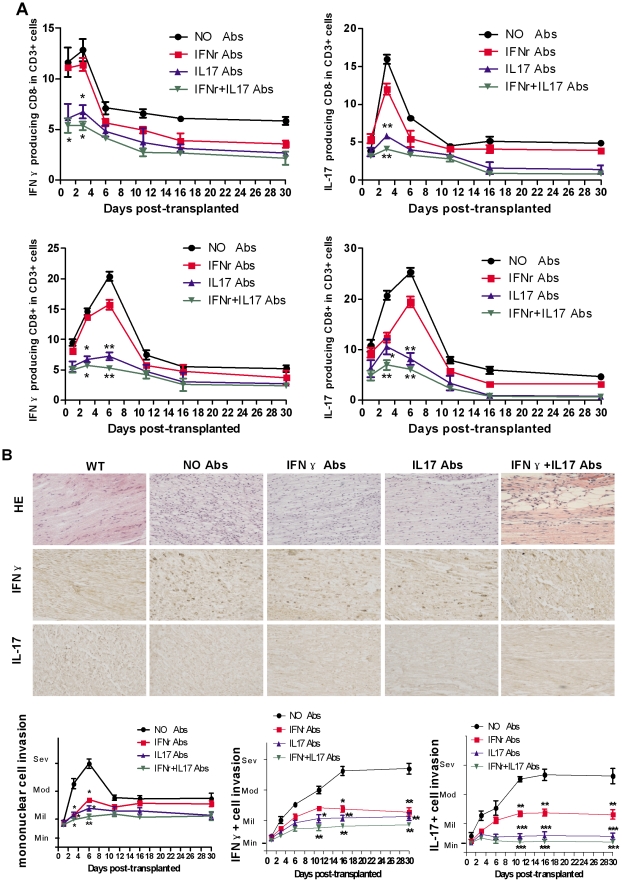
Xenotransplant after using IFN-γ and IL-17 neutralizing antibodies respectively. (A) Spleens were collected at each time point (1, 3, 6, 11, 16, and 30 days) from xenotransplanted mice with or without neutralizing antibodies (single used and mixed used) treatment, and used for flow cytometry analysis. The levels of IFN-γ producing CD3+CD8− T cells and IL-17 producing CD3+CD8− T cells from recipients with IL-17 neutralizing antibody and mixed used antibodies significantly decreased at day 3 compared with the control group. The levels of IFN-γ producing CD3+CD8+ T cells and IL-17 producing CD3+CD8+ T cells from recipients with IL-17 neutralizing antibody and mixed used antibodies significantly decreased at day 3 and day 6 compared with the control group. (B) Tissues surrounding the xenografts were used to do Immunohistochemical staining to measure the expression of IFN-γ+ and IL-17+ cells. We observed that mononuclear cells invasion significantly decreased at day 3 and day 6; and IFN-γ and IL-17 expression decreased in the recipients with IFN-γ and IL-17 neutralizing antibodies single used and mixed used from day 11 to day 30. *, p<0.05; **, p<0.01; *** p<0.001. (We performed this experiment twice).

In order to investigate the role of IFN-γ and IL-17 in peripheral nerve xenotransplantation, we blocked IFN-γ or (and) IL-17 by using neutralizing antibodies before transplantation of rat nerve into the mice. The sera levels of IFN-γ and IL-17 at day 6 were statistically lower in the xenograft recipients with IFN-γ and IL-17 neutralizing antibodies single and mixed used compared with the recipients without antibodies. The levels of both cytokines returned to values of recipients without antibodies by day 11 after transplantation. There was no significant difference between the groups of antibody single used and mixed used. At the same time, we set up another experiment by injection of the neutralizing antibodies one day after xenotransplantation in which we got the consistent outcome that the animals injected the neutralizing antibody before xenotransplantation (Information S1). The levels of IFN-γ producing CD3+CD8+ T cells and IL-17 producing CD3+CD8+ T cells decreased at day 3 and day 6, IFN-γ producing CD3+CD8− T cells, and IL-17 producing CD3+CD8− T cells decreased at day 3 in the recipients with IL-17 neutralizing antibody and mixed used antibodies compared to the levels of recipients without antibodies. Although the level of IFN-γ and IL-17 was lower in the group with mixed used antibodies than the group of IL-17 antibody single used, there was no significant difference between these two groups (Fig. 5A). We observed scattered immunoreactivity for both IFN-γ and IL-17 in the infiltrating mononuclear cells, and found that mononuclear cells invasion decreased at day 3 and day 6; IFN-γ and IL-17 expression decreased in the recipients with IFN-γ and IL-17 neutralizing antibodies single used and mixed used from day 11 after transplantation (Fig. 5B).

## Discussion

Nerve injuries causing a segmental loss require nerve grafting. However, this procedure often does not provide satisfactory results [31]. The use of revascularized cutaneous nerve autografts, allografts, or artificial nerve grafts may offer a solution to nerve segmental loss as a conduit for nerve regeneration. However, all of these strategies contain problems that limit their clinical use [32]. Based on these limitations, nerve xenografts provide a readily accessible alternative strategy. Compared to autografts and allografts, xenografts can induce stronger rejection. However, the peripheral nerve has decreased antigenicity compared to skin, tendon, and some other organs, suggesting it is useful for continued study as a tissue source for nerve grafts. The mechanisms of peripheral nerve xenograft rejection remain complicated and unclear. For instance, the role of Th17 and any regulatory mechanisms among different Th cell subsets during xenograft rejection are unknown. We observed dynamic temporal changes in cellular expression of IFN-γ, IL-17 and IL-4 during acute rejection after peripheral nerve xenotransplantation. The critical findings of this study demonstrate that mononuclear cells significantly infiltrate in and around the nerve grafts and these cells can express either IFN-γ or IL-17. This expression also increases in serum and spleen during acute rejection of these peripheral nerve xenotransplants. IFN-γ and IL-17 significantly decreased in sera and tissue surrounding the xenografts after IFN-γ and IL-17 neutralizing antibodies treatment Also we found that IFN-γ and IL-17 decreased in spleen after using IL-17 neutralizing antibody and using mixed antibodies. It appeared that both CD4+ and CD8+ T cells were involved in the rejection process and Th17 along with Th1 cells may contribute to the development of acute rejection in mice with peripheral nerve xenotransplant.

It has been demonstrated that cellular immunity, especially T cells, plays a vital role during organ xenotransplant rejection [8]–[9], [33]–[34]. In addition to direct killing activity by cytotoxic T lymphocytes, xenograft rejection can occur through T-cell-mediated mechanisms, including cytokine production, recruitment and activation of other cytotoxic cells, as well as by providing help for B cells that produce xenoreactive antibodies [35]. T lymphocytes are activated after contact with APCs presenting antigens expressed by Schwann cells, and produce a set of cytokines including IFN-γ, IL-2, IL-4, and IL-6. Our demonstration of increased IFN-γ expression in this study correlates well with prior work indicating that cellular immune responses and Th1 cells play a critical role in nerve xenograft rejection. Although IL-4 producing CD3+CD8−T cells and IL-4 producing CD3+CD8+T cells decreased at day 1 and day3, and increased from day6 in the xenografts recipients compared to the controls, there is no significant difference between these two groups, which suggested that Th2 cells may not play a critical role during the peripheral nerve xenotransplantation and the changes of Th2 cells might be due to the changes of Th1 cells.

The implication that following transplantation, CD8+ T cells can mount an effective immune response independently of help from CD4+ T cells, has recently gained support from studies of CD8+ T cell responses to pathogens [36]–[39]. Kreisel *et al*. reported that mouse non-hematopoietic cells activated alloreactive CD8+ T lymphocytes *in vitro* and *in vivo* [40]. The CD8+IL-17-producing T cell subpopulation is also important for effector functions during the elicitation of contact hypersensitivity [41]. CD8+IL-17-producing T cells are stimulated by IL-23 but inhibited by IL-12, implicating that different mechanisms regulate the development and function of CD8+ IFN-γ T cells and CD8+IL-17 T cells [41]. Our data indicated that IFN-γ and IL-17 produced by CD8+ T cells increased in the spleen from day 1 to day 6 post xenotransplantation. This implies that CD8+ IFN-γ T cells and CD8+IL-17 T cells both played a role during peripheral nerve acute rejection. However, the regulatory mechanisms between these two cell populations needs to be further studied.

Th17 cells expressing retinoic acid-related orphan receptor γt(RORγt) play a crucial role in the development of autoimmunity and transplant rejection, by producing pro-inflammatory cytokines IL-17, and to a lesser extent, IL-23 and IL-6 [42]. Some studies have demonstrated that Th17 and IL-17 contribute to rejection during heart, lung, liver, and other organ allotransplantation [12], [20], [43]–[45]. The role of Th17 and IL-17 in xenotransplantation rejection remains unknown. However, our findings demonstrated that serum levels of IL-17 and spleen levels of CD3+CD8− IL-17-producing cells increased in mice during acute rejection after peripheral nerve xenotransplantation. Furthermore, we found that IL-17 levels increased in tissue surrounding the xenografts, which might confirm that production of secreted IL-17 was enhanced not only systematically, but locally as well. IL-17 producing T cells significantly decreased in serum, spleen and tissues surrounding the xenografts after single and mixed using IFN-γ and IL-17 neutralizing antibody. These results suggest that Th17 cells, as well as Th1 cells, play a key role in the pathophysiology of nerve xenograft rejection.

There are controversial conclusions regarding the relationship between Th1 and Th17 cells. For instance, a lack of one of these cytokines *in vivo* appears to promote a response dominated by the other. IFN-γ-deficient mice develop an elevated Th17 response and exacerbated disease [14], [23]–[24]. Conversely, IL-17 deficiency may permit emergence of Th1 as an effector response [23]. Other findings underscore the role of the tissue in affecting the site where inflammation occurs, and provide evidence that Th1 and Th17 cells are not always functionally antagonistic, but may in fact collaborate to accomplish some effector functions [25]–[26]. It has become clear that the relationship between the Th1 and Th17 lineages is much more intertwined and complex than was initially appreciated, although there is little data describing any relationship between Th1 and Th17 cells during xenotransplant rejection. Based upon the results from this study, we propose that IFN-γ and IL-17 producing CD3+CD8− cells do not precisely function in parallel during acute rejection. Spleen CD3+CD8− Th1 cells increased from day 1 to day 6, and appeared earlier than the increase in CD3+CD8− Th17 cells from day 3 to day 6. Although spleen and sera changes in xenotransplanted animals returned to control levels by day 11 posttransplant there was a temporal inconsistency between sera and spleen for IL-17. One possibility for this difference could be the fact that IL-17 expressing cells differ between spleen and blood. For example, increased expression of IL-17 by neutrophils, macrophages, or other innate immune cells in blood could explain the increase in sera by day 1 post-xenotransplant whereas the spleen levels of IL-17 are a reflection of primarily lymphocyte expression. Another possibility is that sera concentrations of IL-17 were contributed to primarily by CD3+CD8+ cells in the spleen as these increased by day 1. Any regulatory relationships between Th1 cells and Th17 cells during acute rejection as well as the identity of all immune cells secreting IL-17 requires further study.

Our data demonstrates that both IFN-γ and IL-17 producing CD4+ and CD8+ T cells increase in a temporal fashion during acute nerve xenograft rejection. The rejection significantly decreased after single and mixed using IL-17 and IFN-γ neutralizing antibodies. This suggests that not only Th1 cells, but also Th17 cells contribute to the process of rejection. A better understanding of the ways in which Th1 and Th17 cellular responses interact, is essential for understanding disease pathogenesis and for determining appropriate therapeutic methods for organ xenotransplantation. Further studies may provide new targets for preventing xenograft rejection and promoting regeneration of nerve xenografts.

## Supporting Information

Information S1 A.Sera from collected blood at each time point (1, 3, 6, 11, 16, and 30 days) from xenotransplanted mice with IFN-γ and IL-17 neutralizing antibody single and mixed used were used for ELISA analysis. The sera levels of IFN-γ and IL-17 at day 6 were statistically lower in the xenograft recipients with IFN-γ and IL-17 neutralizing antibodies single and mixed used compared with the recipients without antibodies. The levels of both cytokines returned to values of recipients without antibodies by day 11 after transplantation. B. We performed single and mixed antibody injection at Day 1 after xenotransplantation. Sera from collected blood at each time point (3, 6, 11, 16, and 30 days) from xenotransplanted mice were used for ELISA analysis. There was no significant difference of sera levels of IFN-γ and IL-17 between the groups injected antibody before xenotransplantation and after xenotransplantation. *, p<0.05; **, p<0.01; *** p<0.001. (We performed this experiment twice).
(TIF)
