# Supplementary figures and images for: A Crucial Role of IL-17 and IFN-γ during Acute Rejection of Peripheral Nerve Xenotransplantation in Mice
Source: PLoS One. 2012 Mar 30;7(3):e34419. doi: 10.1371/journal.pone.0034419 (PMC3316676; doi:10.1371/journal.pone.0034419)

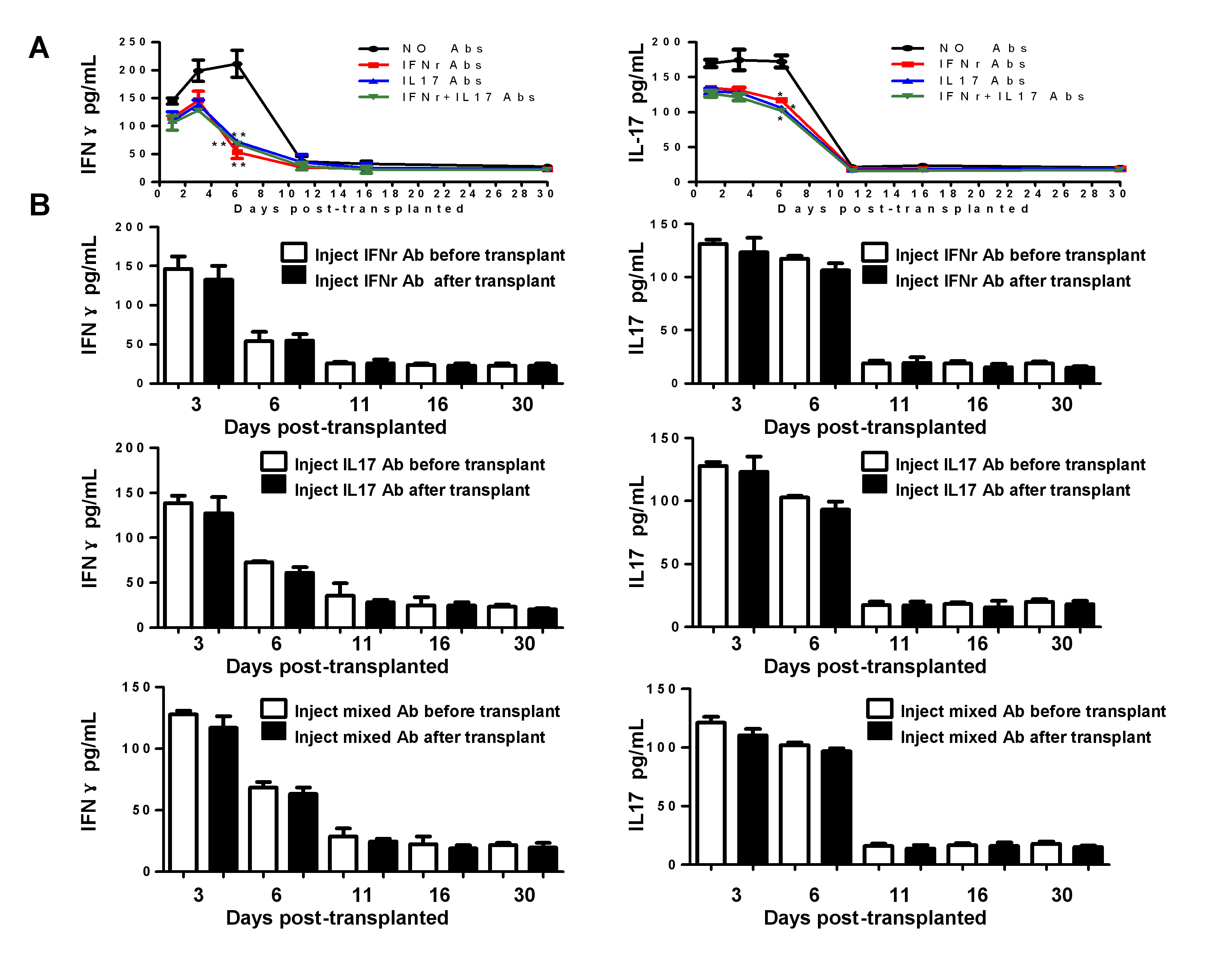

Supplement: Information S1 — A.Sera from collected blood at each time point (1, 3, 6, 11, 16, and 30 days) from xenotransplanted mice with IFN-γ and IL-17 neutralizing antibody single and mixed used were used for ELISA analysis. The sera levels of IFN-γ and IL-17 at day 6 were statistically lower in the xenograft recipients with IFN-γ and IL-17 neutralizing antibodies single and mixed used compared with the recipients without antibodies. The levels of both cytokines returned to values of recipients without antibodies by day 11 after transplantation. B. We performed single and mixed antibody injection at Day 1 after xenotransplantation. Sera from collected blood at each time point (3, 6, 11, 16, and 30 days) from xenotransplanted mice were used for ELISA analysis. There was no significant difference of sera levels of IFN-γ and IL-17 between the groups injected antibody before xenotransplantation and after xenotransplantation. *, p<0.05; **, p<0.01; *** p<0.001. (We performed this experiment twice). (TIF) [file pone.0034419.s001.tif]
